# Advances in Wound Healing: A Review of Current Wound Healing Products

**DOI:** 10.1155/2012/190436

**Published:** 2012-03-22

**Authors:** Patrick S. Murphy, Gregory R. D. Evans

**Affiliations:** Aesthetic and Plastic Surgery Institute, University of California Irvine Medical Center, 200 S. Manchester Avenue, Suite 650, Orange, CA 92868, USA

## Abstract

Successful wound care involves optimizing patient local and systemic conditions in conjunction with an ideal wound healing environment. Many different products have been developed to influence this wound environment to provide a pathogen-free, protected, and moist area for healing to occur. Newer products are currently being used to replace or augment various substrates in the wound healing cascade. This review of the current state of the art in wound-healing products looks at the latest applications of silver in microbial prophylaxis and treatment, including issues involving resistance and side effects, the latest uses of negative pressure wound devices, advanced dressings and skin substitutes, biologic wound products including growth factor applications, and hyperbaric oxygen as an adjunct in wound healing. With the abundance of available products, the goal is to find the most appropriate modality or combination of modalities to optimize healing.

## 1. Introduction

The field of wound care seemingly contains as many different treatment options and modalities as the number of practitioners caring for wounds. While many clinicians rely on and obtain good results with older “tried and true” treatments, there continues to be a constant flow of new products and technologies to add to the wound care armamentarium. Some of these products are updated and improved variations of previous treatments, while others are the result of entirely new fields of study. As with any new product, oftentimes the race to introduction into clinical use precedes adequate controlled study, and the efficacy is then defined by clinical experience. This can lead to unanswered questions regarding appropriate use and indications.

 This paper will discuss several new technologies in burn and wound care. Silver dressings are time honored in wound care, but new forms of delivery aim to increase the efficacy while minimizing side effects. We will also review some of the latest literature on emerging bacterial resistance to these products. Negative pressure wound devices are relatively new in wound care treatment, and their indications are continually expanding to encompass aspects of wound management that previously had very few options. Advanced wound dressing products can help alter the wound environment to optimize healing conditions. With the advent of biosynthetics and tissue engineering, skin substitutes are being created that not only provide novel effective temporary coverage of wounds, but are also changing the paradigm of wound management. By supporting the wound with growth factors and biologic substances, we can help augment or modulate the wound healing process itself. And finally hyperbaric oxygen treatment can provide additional assistance to the above wound healing modalities, especially in chronic wounds not responding to other treatment.

## 2. Silver

The use of silver to prevent and treat infection is both one of the earliest forms of wound care, documented as early as 69 BC, and one of the latest technologies in the realm of antimicrobial prophylaxis. Because silver does have such a favorable broad-spectrum coverage, especially in antibiotic-resistant organisms, with little significant toxicity, there have been a number of new silver-containing wound products developed to capitalize on its wound healing benefits while tailoring the delivery to the most effective means with the fewest side effects.

 Regardless of the nature of the many silver-containing products currently available, elemental silver requires ionization for antimicrobial efficacy [1]. The highly reactive charged silver ion (Ag^+^) reacts by binding to negatively charged particles such as proteins, DNA, RNA, and chloride ions. While this is responsible for its antimicrobial properties, it also complicates delivery as the silver ions are readily bound to proteins and chloride in the wound bed fluid [2]. Many delivery systems exist, with the key to the most effective product being one that can maintain an adequate concentration of silver with long enough residual activity.

 Introduced in 1968, silver sulfadiazine (Flammazine, Silvadene) is silver complexed to various glycols and alcohols and combined with an antibiotic, sulphadiazine [3]. While this combination provides a theoretical advantage by including an additional mechanism of action from the antibiotic, it has been shown to have a higher rate of resistance compared with silver nitrate (1% versus 0.5%) [4], as well as impaired reepithelialization, pseudoeschar formation, and bone marrow toxicity from the propylene glycol [5].

 Nanocrystalline silver dressings were developed and introduced in the late 1990s and are the latest forms of silver wound dressings. These products were designed to overcome some of the shortcomings of previous silver dressings. The typical products currently in use contain two layers of high-density polyethylene net sandwiching a layer of rayon/polyester gauze [6]. The outer layer is coated with a nanocrystalline (<20 nm), noncharged form of silver (Ag^0^), and the inner layer helps maintain a moist environment for wound healing. Because the noncharged silver is less reactive with negatively charged particles in the wound, it is deactivated much more slowly and provides an initial large bolus of silver followed by a sustained release into the wound. Aside from a more consistent therapeutic dose of silver, an additional benefit of nanocrystalline dressings is less frequent dressing changes, on the order of days as compared to standard, twice daily dressing changes for silver sulfadiazine and up to twelve times per day for silver nitrate. This decreases patient discomfort as well as provides less disruption to the healing wound bed.

 Silver has a very broad spectrum of microbial coverage, including yeast, fungi, mold, and even antibiotic-resistant bacteria such as methicillin-resistant *Staph aureus* (MRSA) and vancomycin-resistant enterococci (VRE) when used at appropriate concentrations. Silver is a bactericidal material that kills on contact by inhibiting the respiratory chain at the cytochrome level, as well as, interfering with electron transport [6], denaturing nucleic acids, inhibiting DNA replication, and altering cell membrane permeability [5].

 While bacterial resistance to silver is exceedingly low, it has been reported in the literature since 1975 [7, 8]. This has mostly come from burn units using silver salts as antiseptic agents. Identified silver-resistant strains include *E. coli, Enterobacter cloacae, Klebsiella pneumoniae, Acinetobacter baumannii, Salmonella typhimurium, *and *Pseudomonas stutzeri* [7–10]. In fact a silver-resistant *Salmonella* species caused the closure of the burn unit at Massachusetts General Hospital after septicemia and death in three patients [11].

The challenge is to keep a high enough concentration of agent with a long enough residual activity to prevent developing resistance. The suggested concentration of silver in any preparation is greater than 30 to 40 mg/L to be effective. Traditional silver preparations, silver nitrate and silver sulfadiazine, both are able to provide a high enough initial concentration (3176 mg/L and 3025 mg/L, resp. [4]), but have little to no residual activity. Silver nitrate must be applied twelve times per day to maintain effectiveness. Nanocrystalline silver technology is able to more consistently maintain adequate concentrations (at least 70 mg/L) with good residual activity, keeping levels elevated over longer periods of time.

 To demonstrate the effectiveness of these dressings, Yin et al. [12] compared between nanocrystalline silver, silver sulfadiazine, and silver nitrate by inoculating each with 10^7^ CFU of *S. aureus*. After one hour the nanocrystalline dressing had fewer than 100 organisms remaining, whereas the silver nitrate took 4 hours and silver sulfadiazine took 6 hours to achieve the same result. Wright et al. [13] showed that nanocrystalline dressings killed MRSA in 30 minutes, whereas other silver preparations had no effect. They obtained similar results with VRE. Numerous other studies have shown similar results, with overall time-for-time higher MRSA clearance rates with nanocrystalline silver versus silver sulfadiazine [14]; faster wound healing times, reduced cellulitis, and reduced need for antibiotics with nanocrystalline silver [15]; less burn wound sepsis again with nanocrystalline silver [16]. Though these results do appear very promising, Khundkar et al. [6] express a word of caution. In their recent literature review comparing nanocrystalline silver to other silver preparations, only 1 of 31 articles was rated as Level of Evidence 1 (randomized controlled trial of sufficient size for narrow confidence interval), with the majority of articles LOE 5 (expert opinion or based on bench research).

Silver concentrations lethal to bacteria can also cause damage to healthy cells. In vitro studies on silver nitrate show a negative impact on fibroblasts [17]. Studies on nanocrystalline silver show inhibition of keratinocyte growth [18] and delay in reepithelialization [19], leading to recommendations against using topical silver as a dressing on cultured skin grafts. Further in vitro studies have shown nanocrystalline silver to be specifically toxic to cultured skin substitutes, with cytotoxic effects occurring within 1 day. However, in vivo exposure of these cultured cells to nanocrystalline silver for up to 1 week did not impair wound healing.

 Overall silver is a very effective modality for prevention or treatment of infection over a wide variety of bacteria, viruses, fungi, and molds, with few side effects. Though there are some reports of developing resistance, with continued appropriate use it should remain effective against even multi-antibiotic-resistant microorganisms.

## 3. Negative Pressure Wound Devices

The first paper describing the use of a negative pressure wound device (NPWD) in a series of patients was published thirteen years ago [20]. While the device itself has undergone some minor advances in technology, the most significant changes have come from the indications for use. Advances in the actual device include smaller size, allowing for portable units for home use, increased ability to remove large amounts of fluid, the ability to instill fluids in the wound for continuous irrigation, refinements in the foam with more consistent pore sizes, different sponge materials including silver, and increased safety and alarm systems [21].

 Acute wounds are now more frequently being treated with NPWD closure. In patients with significant comorbidities or other serious injuries, NPWDs can be used in large soft-tissue injuries, contaminated wounds, and wounds with compromised tissue [21]. The protocol is altered to include more frequent dressing changes with serial debridement as necessary. There are several descriptions of the sponge being placed over vital structures such as vessels, nerves, viscera, or even heart or lung [21, 22]. Ideally, muscle or soft tissue should be placed between the structure and the sponge, but if this is not possible Vaseline or silicone mesh should be used. This allows simplified wound closure in critical patients allowing the focus to shift to stabilizing the patient for later definitive reconstruction with flaps. One discovery that has come about by treating acute wounds in this manner is that the overall volume and dimensions of the wound tend to decrease with NPWD closure, possibly allowing a less complex reconstruction than would initially be required [22]. Many complex wounds in stable patients are now being temporized with NPWDs to allow a simpler reconstruction.

 Enterocutaneous fistulas were initially a contraindication for NPWD closure. Some of these patients are now being treated with NPWDs with good results. In published studies, approximately two-thirds of forty reported patients had closure of the fistula with the device, albeit the majority of these patients had low-output fistulas [22]. Trauma patients undergoing damage-control laparotomy and abdominal compartment syndrome patients are also being temporized by allowing an open abdomen with the NPWD in place. This provides temporary coverage and, more importantly, provides a mechanism for removal of intraabdominal contamination and exudates while relieving visceral edema [23, 24]. These patients traditionally required large hernia repairs with mesh, but with the NPWD, a high percentage are able to be primarily closed.

 Traumatic orthopedic injuries have seen bold advances with the advent of NPWD closure. Significant extremity wounds were previously treated with wide debridement of any questionable tissue with free flaps being required for immediate coverage. With the NPWD, debridement is targeted to nonviable tissue, with the device providing a sealed, protected, and moist environment that actively removes edema and hematoma, which increases perfusion and maximizes salvage of the zone of stasis. Serial debridements are performed, with definitive reconstruction occurring in a stable wound on an elective basis [25]. Exposed tendon, bone, or joints are no longer a contraindication as granulation tissue will form over these structures, allowing a bed for skin grafting if necessary [26, 27]. Even inert material such as orthopedic hardware, vascular synthetic graft, or synthetic mesh will develop granulation tissue with the NPWD [22]. These structures are ideally reconstructed with protective flaps, but skin grafting over this granulation tissue is an option in patients that are not flap candidates.

## 4. Advanced Dressings

While plain gauze is still the most commonly used dressing in hospitals today, new wound understanding and technology have produced advanced products that help the body achieve the ideal moist, warm, protected wound healing environment (Table 1). Plain gauze certainly has its place as it is inexpensive, readily available, and appropriate for a large number of wounds. Impregnated gauze improves upon this by adding zinc, iodine, or petrolatum to help prevent desiccation and provide nonadherent coverage.

 The process of autolysis is important in wound care. If an occlusive dressing is provided as a barrier to the outside environment, the body's own phagocytic processes will provide debridement of wounds. These products range from occlusive films such as Tegaderm, which are permeable to air and water vapor, but impermeable to fluid and microorganisms to hydrocolloids such as DuoDERM, which are also occlusive but provide absorption of exudates in addition to maintaining a moist environment for autolysis. For heavily exudative wounds, there are a range of absorptive products including various hydrophilic foam dressings, hydrogels, hydrofibers, and alginates, which can absorb up to 20 times their weight. Though these products are more expensive than traditional gauze, recent studies seem to indicate an overall cost savings due to decreased labor costs: advanced dressings typically are changed every 1 to 3 days, as opposed to gauze, which is often changed multiple times per day [28]. Additionally, faster healing times from advanced dressings mean an overall decreased treatment period.

## 5. Skin Substitutes

 The advances in temporary and permanent coverage of wounds have made significant gains with advancing technology in biomaterials and tissue engineering. Burn wounds are the major indication for these products. With advances in burn resuscitation and critical care management, more patients with significant body surface area burns are surviving, leading to the issue of coverage of large wounds. Autograft is currently the preferred option, but in many instances there is an insufficient amount of tissue available for grafting, or the patient's condition precludes the use of autograft. Allografts and xenografts can provide a temporary coverage option, but they come with issues regarding rejection, and possible disease transfer, availability, as well as cultural and ethical considerations.

Bioengineered skin substitutes, both biosynthetic skin substitutes and cultured autologous engineered skin, are available to provide temporary or permanent coverage, with the advantages of availability in large quantities and negligible risk of infection or immunologic issues. The main limitation of these products is their expense. We will briefly discuss currently available products and further discuss some of these products that may confer an advantage over autologous tissue in terms of potential for wound healing in chronic wounds.

Biobrane is a temporary dressing composed of knitted nylon mesh bonded to a thin silicone membrane and coated with porcine polypeptides [29]. It is used in clean superficial and middermal depth burns or as coverage for donor sites in split-thickness skin grafting. Studies have shown it to be as efficacious as silver sulfadiazine in wound healing without the frequency of dressing changes [30].

TransCyte is a biosynthetic dressing of a semipermeable silicone membrane on a nylon mesh coated with porcine collagen and newborn human fibroblast cells [29]. It is used as a dressing in superficial burns that do not require skin grafting, or as a temporary cover for excised burns prior to grafting. Several studies have shown it to be superior to antibiotic creams or silver sulfadiazine in terms of healing time, infections, and scar formation, especially on facial burns [31, 32].

Dermagraft contains neonatal fibroblasts on a bioabsorbable polyglactin mesh. The fibroblasts produce dermal collagen, glycosaminoglycans, growth factors, and fibronectin to support wound healing [30]. It is a temporary or permanent cover used for excised burn wounds as well as venous ulcers and pressure ulcers [29]. Results show it to be comparable to allograft for wound infection, healing time, exudates, and graft take, with higher patient satisfaction [33, 34].

Apligraf is composed of an epidermal layer of allogeneic neonatal keratinocytes and fibroblasts from neonatal foreskin on bilayered type I bovine collagen [29, 35] that is used as an adjunct covering to autograft, providing accelerated healing times [30]. It is also used alone in chronic wound ulcers, showing increased healing times when compared to controls [36].

Integra is a semibiologic bilayered dressing composed of a matrix of type I bovine collagen, chondroitin-6-sulfate, a glycosaminoglycan from shark cartilage, under a temporary silicone epidermal sheet [29, 37]. The pore size (70–200 *μ*m) is designed to allow migration of the patient's own endothelial cells and fibroblasts. As the wound heals, the silicone sheet is removed and a thin autograft is grafted onto the neodermis to complete the wound coverage. It is indicated for excised deep partial- and full-thickness burn wounds. Additionally, in nonburn wounds it is used in complex traumatic soft tissue reconstruction over exposed tendons, joints, and bone, as well as wounds from vascular and pressure ulcers [38]. A study with 10-year followup shows excellent cosmesis, with higher patient satisfaction than autologous skin grafting alone and with excellent mobility when placed over joints [35]. In children involved in the study, the product was able to grow with the child. Jeng et al. [39] describe their 7-year experience with 44 patients using Integra to cover soft tissue defects over exposed bone, tendon, and joints, often using multiple serial layers of Integra to fill in large depressions. They have had overall good results and feel they have potentially saved several extremities that otherwise would require amputation.

## 6. Growth Factors and Biologic Wound Products

Biologic wound products have been an area of tremendous growth as our understanding of the details of the wound healing response has increased. In normal wound healing there is an orderly, predictable sequence passing through the inflammatory, proliferation, and remodeling/maturation phases. This process is driven by numerous cellular mediators including eicosanoids, cytokines, nitric oxide, and various growth factors. The field of biologic wound products aims to accelerate healing by augmenting or modulating these inflammatory mediators. While the majority of investigations on these substances are small laboratory studies, there are some clear benefits seen in clinical investigations.

 Eicosanoids are arachadonic acid metabolites including prostaglandins, prostacyclines, thromboxane, and leukotrienes. They primarily affect the early stages of wound healing including initial vasoconstriction and later vasodilation, vascular permeability, and inflammatory cell chemotaxis and adhesion. The most well known is prostaglandin E_1_ which inhibits platelet and neutrophil activation, reduces blood viscosity, stimulates tissue plasminogen activator production, and causes vasodilation by relaxing vascular smooth muscle [38]. Because of this ability to modulate inflammation and vasodilate, its use in chronic vascular ulcers has been evaluated with some significant decrease in size and healing time compared to controls [38, 39].

 Cytokines regulate inflammation by influencing hematopoietic cells and include chemokines, lymphokines, monokines, interleukins, colony-stimulating factors, and interferons. Several of these have been studied. Interleukin-1, which stimulates most cells in the wound environment, was tested in pressure ulcer patients with equivocal results [40]. Granulocyte/macrophage colony-stimulating factor (GM-CSF) has been most extensively studied. Its effects are to stimulate neutrophils, macrophages, keratinocytes, and fibroblasts and increase VEGF production, rendering it a very promising molecule in wound healing [41]. There have been encouraging results in a prospective randomized control study involving patients with venous stasis ulcers [42], as well as studies on diabetic-foot ulcers [43].

 Growth factors stimulate mainly fibroblasts and keratinocytes via transmembrane glycoproteins [44]. They have been studied more than any other biologic wound supplement. They are divided into five superfamilies, the most known being the platelet-derived growth factors. Recombinant PDGF was studied in a series of 118 patients with diabetic-foot ulcers by Steed et al. [45]. Patients were treated with rhPDGF or placebo for up to 20 weeks in this prospective randomized double-blind study. The rhPDGF group showed a statistically significant higher percentage of patients that achieved wound healing, 48% versus 25%, as well as a greater reduction in wound size. This study helped lead to FDA approval of rhPDGF for diabetic ulcers, which is now known as becaplermin, with its trade name being Regranex. It is the only current FDA-approved product in the growth factor family. Additional studies have confirmed increased odds of wound healing and decreased rates of amputation in diabetic foot ulcers [46], as well as accelerated wound healing in abdominal wound separation [47] and irradiated wounds [48].

## 7. Hyperbaric Oxygen

Hyperbaric oxygen is a treatment modality that has been used as an adjunct in wound healing for 40 years. It involves placing the patient in a sealed chamber where 100% oxygen is pressurized to between 1.5 and 3 atmospheres absolute (ATA) for 60 to 120 minutes over a course of multiple treatments. Originally designed for use in decompression illness in deep sea divers, it has indications for use for carbon monoxide poisoning, crush injuries, compartment syndrome, acute traumatic ischemia, ischemia-reperfusion injury, radiation injury, compromised skin grafts, infections with anaerobic organisms, and refractory osteomyelitis. In addition, there are some specific indications for HBO therapy in chronic wounds [49].

Hyperbaric oxygen has few absolute contraindications. Reactive airway disease, untreated pneumothorax, and concurrent chemotherapy are absolute contraindications due to air trapping, potential for tension pneumothorax, and increased morbidity with chemotherapy [49]. Adverse reactions include otic or sinus discomfort, claustrophobia, and neurologic oxygen toxicity seen at high pressures. This can be reduced by providing air breaks during treatment.

 The mechanism of action of hyperbaric oxygen is not clearly understood, but several studies are currently ongoing. Initial theories focused on increases in oxygen availability at the tissue level [49]. The increased atmospheric pressure increases arterial oxygen pressure (PaO_2_), which in turn causes vasoconstriction. This vasoconstriction on the arterial end reduces capillary pressure, which promotes fluid absorption into the venous system thereby reducing edema, as well as causing an increase in hyperoxygenated plasma to the tissues. This effect typically lasts for several hours after the treatment has finished [50]. Tissue repair processes such as collagen elongation and deposition and bacterial killing by macrophages are dependent upon oxygen, so increased levels, especially in wound areas that already have impaired perfusion, serve to facilitate wound healing.

 On a molecular level, recent studies have focused on the effects of hyperbaric oxygen on neovascularization of diabetic wounds. Angiogenesis refers to the ingrowth of new vessels into a wound from the surrounding tissue. Vasculogenesis is the process whereby progenitor stem cells differentiate and reform a vascular network within a wound [51]. These processes are impaired in the diabetic patient, but evidence suggests that hyperbaric oxygen can help improve these pathways.

 Neovascularization in wounds is dependent upon two main processes [52]. First-endothelial progenitor cells (EPCs) and other stem cells are mobilized from the bone marrow into the circulation. Elevated systemic levels of these cells must then interact with appropriate local wound factors and cytokines, namely, stromal-cell-derived factor (SDF)-1*α*, in order to stimulate vasculogenesis. Diabetic patients have been shown to have decreased numbers and impaired function of bone-marrow-derived EPCs [53, 54], and impaired EPC recruitment [55]. Hyperbaric oxygen is shown to stimulate EPCs and stem cell release from bone marrow both by increased cell proliferation within the marrow, as well as by rapid mobilization via matrix metalloprotease mechanisms [56–58]. This appears to be mediated through nitric oxide pathways [57]. The stimulation of nitric oxide pathways by hyperbaric oxygen is also supported by several studies of HBO in ischemia-reperfusion injury [59–61].

 Initial studies on hyperbaric oxygen showed reduced size of chronic lower-extremity wounds in insulin-dependent diabetics [62]. A 2004 Cochrane evaluation of HBO therapy in chronic wounds looked at randomized controlled trials comparing HBO to no-HBO treatment [63]. They found a total of 5 studies that met inclusion criteria. 147 patients in four studies dealt with diabetic-foot ulcers, and the results showed a significant decrease in the rates of major amputations, as well as an increase in the number of wounds that remained healed 1 year posttreatment. The fifth study of 16 patients showed a decrease in the size of venous ulcers at 6 weeks. An additional study comparing lower-extremity wounds treated by HBO, standard wound care, growth factor therapy, or HBO plus growth factor therapy showed a significant increase in healing at 8 weeks in the HBO group compared to the standard care and growth factor groups, with no additional benefit being seen by the HBO plus growth factor group [63].

 More recently the Hyperbaric Oxygen Therapy in Diabetics with Chronic Foot Ulcers (HODFU) study was completed [64]. This randomized, double-blinded, placebo-controlled study compared between Wagner grade 2, 3, or 4 chronic ulcers treated with hyperbaric oxygen or hyperbaric air. This study found statistically significant improvement in wound healing at 1 year with hyperbaric oxygen (52% versus 29%, *P* = 0.03), with even better results in those patients completing >35 sessions (61% versus 27%, *P* = 0.009). In analysis the largest difference in healing rate was seen after 9 months. This corresponds with a previous RCT study showing no significant difference in HBO-treated groups at 6 weeks, but achieving statistical significance in wound healing at 1 year [65]. This suggests that in addition to immediate assistance in healing, hyperbaric oxygen also has a role in long-term wound improvement, perhaps as the full effects of neovascularization are realized. More studies are needed to delineate these mechanisms.

## 8. Conclusion

The field of wound care is ever expanding with advances in technology. While there is still no superior substitute for reconstruction using patients' own tissues and carefully thought-out reconstructive procedures; new products can help facilitate eventual healing by providing prophylaxis against barriers to healing, augmentation of wound healing factors, assistance in temporizing and bridging time to definitive repair, and optimization of the ultimate results of wound reconstruction. Current wound healing products and modalities increase the armamentarium of the wound practitioner to address all aspects of wound care.

## Figures and Tables

**Table 1:** Advanced wound dressings.

Protective dressings	Notes
Gauze	Inexpensive; readily available
Impregnated gauze	Nonadherent; preserves moisture
Antimicrobial dressings	
Antibacterial ointments	Reapply often to maintain moisture
Iodine based	Absorbent; not for use with thyroid disorders
Silver based	Many forms; broad spectrum; low resistance
Autolytic debridement	
Films	Occlusive; allows exchange of gasses
Hydrocolloids	Not for exudative or infected wounds
Hydrogels	Rehydrates to soften dry wounds
Chemical debridement	
Papain/urea	Availability issues in US
Collagenase	Selective debridement
Absorbent dressings	
Foam	Absorbs moderate exudate
Hydrogels	Absorbs minimal exudate
Hydrofibers	Absorbs heavy exudate
Alginates	Absorbs heavy exudate
